# Reducing Personal Exposure to Particulate Air Pollution Improves Cardiovascular Health in Patients with Coronary Heart Disease

**DOI:** 10.1289/ehp.1103898

**Published:** 2012-01-03

**Authors:** Jeremy P. Langrish, Xi Li, Shengfeng Wang, Matthew M.Y. Lee, Gareth D. Barnes, Mark R. Miller, Flemming R. Cassee, Nicholas A. Boon, Ken Donaldson, Jing Li, Liming Li, Nicholas L. Mills, David E. Newby, Lixin Jiang

**Affiliations:** 1 Centre for Cardiovascular Science, University of Edinburgh, Edinburgh, United Kingdom; 2 Department of Epidemiology and Biostatistics, School of Public Health, Peking University, Beijing, People’s Republic of China; 3 National Institute for Public Health and the Environment, Centre for Environmental Health Research, Bilthoven, the Netherlands; 4 Fuwai Hospital and Cardiovascular Institute, Chinese Academy of Medical Sciences, and Peking Medical Union College, Beijing, People’s Republic of China

**Keywords:** air pollution, blood pressure, face mask, heart rate variability, myocardial ischemia

## Abstract

Background: Air pollution exposure increases cardiovascular morbidity and mortality and is a major global public health concern.

Objectives: We investigated the benefits of reducing personal exposure to urban air pollution in patients with coronary heart disease.

Methods: In an open randomized crossover trial, 98 patients with coronary heart disease walked on a predefined route in central Beijing, China, under different conditions: once while using a highly efficient face mask, and once while not using the mask. Symptoms, exercise, personal air pollution exposure, blood pressure, heart rate, and 12-lead electrocardiography were monitored throughout the 24-hr study period.

Results: Ambient air pollutants were dominated by fine and ultrafine particulate matter (PM) that was present at high levels [74 μg/m^3^ for PM_2.5_ (PM with aerodynamic diamater <2.5 µm)]. Consistent with traffic-derived sources, this PM contained organic carbon and polycyclic aromatic hydrocarbons and was highly oxidizing, generating large amounts of free radicals. The face mask was well tolerated, and its use was associated with decreased self-reported symptoms and reduced maximal ST segment depression (–142 vs. –156 μV, *p* = 0.046) over the 24-hr period. When the face mask was used during the prescribed walk, mean arterial pressure was lower (93 ± 10 vs. 96 ± 10 mmHg, *p* = 0.025) and heart rate variability increased (high-frequency power: 54 vs. 40 msec^2^, *p* = 0.005; high-frequency normalized power: 23.5 vs. 20.5 msec, *p* = 0.001; root mean square successive differences: 16.7 vs. 14.8 msec, *p* = 0.007). However, mask use did not appear to influence heart rate or energy expenditure.

Conclusions: Reducing personal exposure to air pollution using a highly efficient face mask appeared to reduce symptoms and improve a range of cardiovascular health measures in patients with coronary heart disease. Such interventions to reduce personal exposure to PM air pollution have the potential to reduce the incidence of cardiovascular events in this highly susceptible population.

Air pollution exposure is an established risk factor for cardiovascular morbidity and mortality (Brook et al. 2010), especially exposure derived from traffic and industrial sources (Laden et al. 2000; Lall et al. 2011; Lipfert et al. 2006; Pope et al. 2002; Sarnat et al. 2008). Acute exposure to combustion-derived particulate matter (PM) is associated with the onset of myocardial infarction and admissions to hospital in survivors of myocardial infarction and has been proposed as a trigger for acute cardiovascular events (Peters et al. 2004; von Klot et al. 2005). Although estimates vary, chronic exposure to air pollution has been estimated to increase all-cause mortality by 2–4% per 10-μg/m^3^ increase in PM (Dockery et al. 1993; Pope et al. 2002), with most deaths due to cardiovascular disease (Miller et al. 2007). The World Health Organization estimates that outdoor urban air pollution results in around 800,000 deaths worldwide each year (United Nations Environment Programme 2006).

In controlled exposure studies, inhalation of PM air pollution affects blood pressure and causes abnormalities in vascular function, coagulation, and myocardial perfusion (Mills et al. 2009). These responses provide a plausible mechanism to explain the observed increase in acute cardiovascular events and cardiovascular mortality after exposure to PM air pollution. However, although acute exposure induces these adverse effects, whether improvements in cardiovascular health can be achieved by interventions targeted to reduce exposure in those living and working in highly polluted urban environments is unclear.

Major environmental health policy interventions can have a substantial impact on the health of populations, as evidenced by major reductions in cardiovascular events after the banning of bituminous coal in Dublin, Ireland, in 1990 (Clancy et al. 2002) and, more recently, with the restriction of smoking in public places (Pell et al. 2008). However, such environmental interventions may be difficult to implement in rapidly developing countries where economic growth is dependent on road traffic and heavy industry (Smith et al. 2009). More practical solutions to reduce individual exposure and protect susceptible persons are urgently required. Therefore, we investigated the effects of a simple face mask intervention to reduce PM air pollution exposure on measures of cardiovascular health in patients with coronary heart disease.

## Methods

*Subjects.* One hundred and two patients were recruited from the Fuwai Hospital, Beijing, China, in March 2009. All patients were nonsmokers and had a history of coronary heart disease. Exclusion criteria were a history of arrhythmia, severe coronary artery disease without revascularization, resting conduction abnormality, digoxin therapy, uncontrolled hypertension, renal or hepatic failure, or an acute coronary syndrome within the previous 3 months. Patients’ medical histories were recorded from the case notes, and baseline anthropometric and biochemical measures were performed on recruitment. All subjects gave their written informed consent, and the study was reviewed and approved by the local research ethics committee.

*Study design.* Subjects attended the Fuwai Hospital or the ChaoYang Hospital in Beijing on two occasions, with at least a week between visits (median time between visits was 9 days), between March and May 2009. Each subject attended the same hospital on each visit. In a prospective randomized open blinded end point (PROBE) crossover study, subjects walked for 2 hr between 0900 hours and 1100 hours along prescribed city center routes [see Supplemental Material, Figure S1 (http://dx.doi.org/10.1289/ehp.1103898)] in Beijing, using a highly efficient face mask on one study visit but not the other (Dust Respirator 8812; 3M, St. Paul, MN, USA). This mask consists of a lightweight polypropylene filter, which is effective at removing airborne PM without affecting ambient gases. The mask has an expiration valve, complies with EN149:2001 FFP1 European Standard (British Standards Institute 2001), and has an assigned protection factor of 4 [i.e., it can be worn in atmospheres containing up to four times the workplace exposure limit (WEL) as defined by the U.K. Health and Safety Executive (2011). [The WEL for respirable carbon particles (carbon black), is 3.5 mg/m^3^ over an 8-hr time weighted average.] Mask use was randomly assigned to the first or second visit using balanced computer-generated randomization. In order to maximize the difference in PM air pollution exposure, subjects wore the mask for 24 hr before the mask study day, in addition to wearing it during the 24 hr study day, and were given instructions to wear the mask at all times while outdoors and as much as possible when indoors. Subjects’ activities after the prescribed walk were not restricted, and they were instructed to continue their normal daily routines.

*Personal pollution exposure and activity monitoring.* Personal air pollutant exposure was determined using monitoring equipment contained within a backpack. Fine particulate matter (PM_2.5_; PM with aerodynamic diameter ≤ 2.5 μm) was determined using a DataRAM monitor (model pDR-1500; Thermo Scientific, Franklin, MA, USA), and particle number was measured using a condensation particle counter (model CPC 3007; TSI Instruments Ltd., High Wycombe, UK). Ambient temperature and relative humidity were recorded using an external sensor (Omegaette® model HH-314; Omega Engineering Ltd., Stamford, CT, USA). Gaseous pollutants were measured using a multigas analyzer with electrochemical sensors for carbon monoxide, sulfur dioxide, and nitrogen dioxide (model X-am 7000; Dräger Safety, Lübeck, Germany). During the prescribed walk, physical activity was measured using global positioning system (GPS) tracking (eTrex Summit HC GPS unit; Garmin, Olathe, KS, USA), and energy expenditure was estimated using activity data and anthropometric data as described previously (Langrish et al. 2009). The estimated PM exposure when wearing the mask was determined based on measurements of mask filter efficacy as described previously (Langrish et al. 2009).

*Background pollution monitoring.* Background exposure was recorded from permanent monitoring stations in the district where the patients walked on the study day (Beijing Municipal Environmental Protection Bureau 2009). Airborne PM was collected onto Teflon filters (Pall Corp., Ann Arbor, MI, USA) in three size fractions: coarse (mean aerodynamic diameter, 2.5–10 μm), fine (0.18–2.5 μm), and ultrafine (< 0.18 μm) using a MOUDI cascade impactor (MSP Corp., Shoreview, MN, USA).

PM mass was determined gravimetrically for each size fraction from the above filters after temperature and humidity conditioning and subsequently analyzed for elemental and organic carbon fractions, metals and cations, nitrate and sulfate anions, and organic matter.

*Chemical and toxicological analysis of collected PM.* Collected PM samples were analyzed for total carbon content, as well as elemental and organic carbon fractions, using the Sunset method (National Institute for Occupational Safety and Health 2003). Metals and cations were determined using inductively coupled plasma mass spectrometry (ICP-MS) after pretreatment with nitric acid. Nitrate and sulfate anions were determined after extraction with water using liquid chromatography paired with ICP-MS. Organic matter was extracted from filters by ultrasonification with toluene and analyzed using gas chromatography/mass spectrometry.

The oxidative potential of PM was assessed using electron paramagnetic resonance (EPR) (Miller et al. 2009). EPR was used to establish oxygen-centered free radical generation from the collected PM in the absence of tissue. Filters for all size fractions were pooled and sonicated in phosphate-buffered saline to give a final concentration of 1 mg particles/mL. Solutions were incubated with the spin-trap 1-hydroxyl-2,2,6,6,-tetramethyl-4-oxo-piperidine (Tempone-H; 1 mM) (Enzo Life Sciences, Exeter, UK), loaded into a capillary tube and assessed at 37°C in an X-band EPR spectrometer (model MS-200; Magnettech, Berlin, Germany) as described previously (Miller et al. 2009). Pyrogallol (100 μM) was used as a positive control, and samples were compared with the National Institute of Standards and Technology (NIST; Gaithersburg, MD, USA) standard reference materials for urban dust (SRM-1649a; 1 mg/mL) and diesel exhaust PM (SRM-2975; 10 μg/mL).

*Electrocardiography and blood pressure monitoring.* Continuous 12-lead electrocardiography (ECG) was assessed with a digital Holter recorder (Lifecard 12; Spacelabs Healthcare Ltd., Hertford, UK) using the Pathfinder automated arrhythmia analysis package (version 8.701, DelMar Reynolds’ Pathfinder Digital; Spacelabs Healthcare Ltd.). Identified arrhythmias were individually inspected and verified or deleted as appropriate, and heart rate variability was assessed using the DelMar Reynolds’ HRV Tools software package as described previously (Langrish et al. 2009). ST segment analysis was performed at the J-point +80 msec in three representative leads (II, V2, and V5) that were analyzed separately for each subject. Maximal ST segment depression and ischemic burden (product of the change in ST segment amplitude and the duration of the recording) were determined for each lead and as a composite (Mills et al. 2007).

Ambulatory blood pressure was measured automatically (model 90217 ultralite ambulatory blood pressure monitor; Spacelabs Healthcare Ltd.) every 15 min during the 2-hr walk and then every 30 min during the day and every hour overnight (i.e., 2200 hours to 0700 hours).

*Symptom questionnaire.* Subjects completed a symptom questionnaire at the beginning of the study day, after the 2-hr walk, and at the end of the 24-hr visit. They were asked to report physical symptoms (e.g., headaches, dizziness, nausea), their perception of the pollution, their perceived workload, and the tolerability of the mask after the prescribed walk using a visual analog scale.

*Data analysis and statistical methods.* In our previous study of healthy volunteers, we demonstrated a difference (mean ± SD) in systolic blood pressure of 7 ± 5 mmHg after a 2-hr walk when a face mask was used (Langrish et al. 2009). Based on the assumption that the effect size in the present study would be considerably smaller because of the use of cardiac medications, we powered the study to detect a 2-mmHg difference in systolic blood pressure, giving a sample size of 101 at 80% power and two-sided *p* < 0.05.

Blood pressure and ECG end points were analyzed by investigators unaware of treatment allocation. All data are expressed as medians (interquartile ranges) or means ± SD unless otherwise stated. Treatment × period (order in which the mask intervention was used) interactions were assessed as described previously (Hills and Armitage 1979), before data were compared using paired Student’s *t*-tests or Wilcoxon matched pairs signed rank test as appropriate. Occurrence of arrhythmias, reported symptoms, and ST segment event frequency, were compared using the chi-squared analysis. All data were analyzed using GraphPad Prism (version 4 for Macintosh; GraphPad Software, San Diego, CA, USA). Statistical significance was taken as a two-sided *p* < 0.05.

## Results

**Figure 1:**
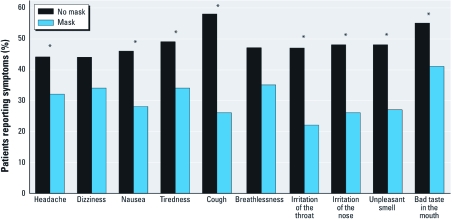
Self-reported symptoms of well-being in the presence or absence of the face mask. *p < 0.05.

*Subjects and face mask intervention.* Ninety-eight patients (87% male; mean age, 62 years) completed the study protocol (Table 1). Four of those originally enrolled did not complete the protocol because of withdrawn consent, cataract extraction, or withdrawal by investigators because of smoking or failure to walk the prescribed route. All subjects tolerated the mask intervention well, scoring the comfort of the mask as 0.64 ± 1.06 on a 0–10 scale (0 represents completely comfortable, and 10, intolerable). The mask intervention reduced self-reported general symptoms (Figure 1) and patients’ perceived effort of work, as well their perception of the level of ambient air pollution (2.46 ± 1.67 vs. 2.73 ± 1.64 on the 0–10 visual analog scale; *p* = 0.03). There were no significant treatment × period interactions for any outcome measure.

**Table 1:** Baseline characteristics of subjects (*n* = 98) completing the study.

Characteristic	Measure
Age (years)	62 ± 7
Male	85
Height (cm)	169 ± 6
Weight (kg)	75 ± 10
BMI (kg/m^2^)	26 ± 3
Risk factors	
Hypertension	79
Diabetes mellitus	45
Stroke	15
Peripheral vascular disease	5
Previous myocardial infarction	68
Previous PCI	60
Previous CABG	38
LV ejection fraction (%; *n* = 31)	62 ± 9
Angina status	
CCS class I	67
CCS class II	31
Seattle angina score (maximum, 500)	387 ± 34
Clinical biochemistry	
Random glucose (mmol/L)	5.5 ± 1.8
Triglycerides (mmol/L)	1.5 ± 0.7
Cholesterol (mmol/L)	3.9 ± 0.9
HDL cholesterol (mmol/L)	1.2 ± 0.3
LDL cholesterol (mmol/L)	2.0 ± 0.8
Medication use	
Aspirin	92
Clopidogrel	17
Warfarin	1
ACE inhibitor or ARB	54
Beta blocker	73
Calcium channel blocker	42
Statin (fibrate, or ezetimibe)	83
Nitrate	45
Other antianginal	5
Diabetic medication	42
Traditional Chinese medicine	19
Abbreviations: ACE, angiotensin converting enzyme; ARB, angiotensin II receptor blocker; BMI, body mass index; CABG, coronary artery bypass graft; CCS, Canadian Cardiovascular Society; HDL, high density lipoprotein; LDL, low density lipoprotein; LV, left ventricle; PCI, percutaneous coronary intervention. Data are mean ± SD, or *n*.	

**Figure 2:**
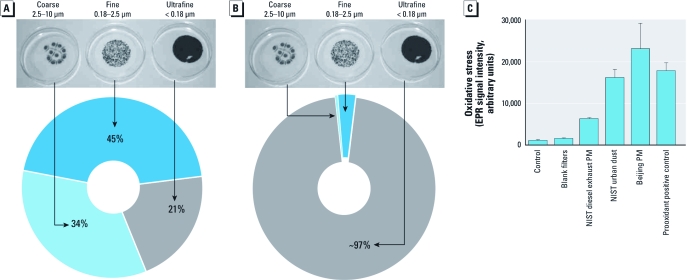
(*A*) Representative filter samples collected showing contribution by mass of the three size fractions averaged over 3 days. (*B*) Estimated contribution of each size fraction collected on filters by particle number. (*C*) Oxidative potential of the collected Beijing PM (1 mg/mL) using EPR to assess oxygen-centered free radical generation, compared with NIST diesel exhaust PM (10 μg/mL) and urban dust (1 mg/mL) and the prooxidant positive control (pyrogallol 100 μM) as described previously (Miller et al. 2009). Data are mean ± SD (*n* = 2–4).

*Air quality and pollutants.* Personal levels of ambient air pollutants were similar on both study days (Table 2), although we predict from previous studies of filter efficacy (97% reduction in particle number) that the PM exposure in the presence of the mask would be reduced from 89 μg/m^3^ and 43,900 particles/cm^3^ to approximately 2 μg/m^3^ and 1,200 particles/cm^3^ (Langrish et al. 2009). Temperature (17.3°C vs. 16.8°C) and ambient relative humidity (30% vs. 35%) were similar on both visits. Airborne PM was predominantly (> 99% by particle number) in the fine and ultrafine fractions (Figure 2) and contained a large amount of organic carbon and high concentrations of polycyclic aromatic hydrocarbons, nitrates, hopanes, and steranes, suggesting that much of the PM was combustion derived and related to traffic sources [see Supplemental Material, Table S1 (http://dx.doi.org/10.1289/ehp.1103898)]. These collected particles were highly oxidizing and generated large amounts of free radicals as detected by EPR, exceeding the signal seen with both the standard reference NIST urban dust material at an equivalent concentration and the intense free-radical–generating oxidant pyrogallol (Figure 2).

**Table 2:** Personal ambient pollution exposures and background pollution levels on days defined according to mask use.

Parameter	Mask	No mask
Personal PM_2.5_ exposure (μg/m^3^)				
Measured		61 (20–88)		89 (25–170)
Estimated		~ 2 (0.6–2.6)		89 (25–170)
Personal particle count (× 10^4^ particles/cm^3^)				
Measured		4.19 ± 1.29		4.39 ± 1.45
Estimated		~ 0.12 ± 0.04		4.39 ± 1.45
Personal temperature (°C)		17.3 ± 5.2		16.8 ± 5.8
Personal relative humidity (%)		30.4 ± 14.0		34.8 ± 18.2
Personal peaks > 1 ppm (number)				
NO_2_		None		None
SO_2_		None		None
CO		5 (2–7.5)		4 (2–8)
Background exposure				
PM_10_ (μg/m^3^)		92 (70–117)		103 (83–180)
SO_2_ (ppb)		38 (29–53)		54 (32–77)
NO_2_ (ppb)		36 (29–42)		36 (32–47)
Abbreviations: CO, carbon monoxide; NO_2_, nitrogen dioxide; SO_2_, sulfur dioxide. Data are mean ± SD or median (interquartile range). Personal monitoring data were collected using portable monitoring equipment during the 2-hr walk. Background data were collected from permanent monitoring stations for the whole 24-hr period. Estimated PM exposure is calculated based on filter efficacy studies where 97% of fresh diesel exhaust PM were removed (Langrish et al. 2009).				

*Effect of the face mask on cardiovascular health.* Over the 24-hr period, the maximal ST segment depression recorded was lower [median (interquartile range), –142 μV (–179 to –110) vs. –156 μV (–202 to –123); *p* = 0.046] when the face mask was used. However, measures of myocardial ischemic burden were similar during the 2-hr walk and over the entire 24-hr period between visits (Table 3).

**Table 3:** Ambulatory blood pressure, heart rate variability during the 2-hr city center walk and the 24-hr study period, and myocardial ischemia measured as ischemic burden, in each individual territory and as a composite according to face mask use.

Walk			24 hr		
Parameter	Mask	No mask	Mask	No mask
Systolic blood pressure (mmHg)		126.9 ± 15.9		128.1 ± 16.5		121.2 ± 11.9		120.8 ± 12.4
Diastolic blood pressure (mmHg)		78.0 ± 9.3		79.5 ± 8.6		73.8 ± 7.2		74.0 ± 7.3
Mean arterial pressure (mmHg)		93.3 ± 9.7*		95.7 ± 10.0		89.8 ± 7.5		90.0 ± 7.9
Heart rate (bpm)		81.5 ± 8.7		81.5 ± 10.1		77.6 ± 11.3		76.7 ± 11.1
LF power (msec^2^)		133 (68–97)		136 (52–227)		81 (40–172)		93 (46–208)
HF power (msec^2^)		54 (27–108)*		40 (20–69)		27 (11–77)		31 (11–68)
LFn (msec)		58.4 (45.6–69.1)*		62.9 (51.1–75.5)		67.2 (55.5–78.0)		71.1 (59.4–81.1)
HFn (msec)		23.5 (18.0–32.4)*		20.5 (13.5–27.9)		21.4 (15.0–31.6)		20.9 (12.7–30.1)
HF:LF ratio		0.418 (0.258–0.712)		0.328 (0.207–0.573)		0.301 (0.190–0.554)		0.306 (0.161–0.492)
pNN50 (%)		1.2 (0.2–2.8)		0.7 (0.0–2.3)		0.5 (0.0–3.1)		0.6 (0.0–2.6)
RMSSD (msec)		16.7 (13.2–22.5)*		14.8 (10.9–19.6)		15.5 (11.0–22.6)		14.4 (10.3–20.3)
SDNN (msec)		59.8 (46.4–79.1)		60.1 (41.0–79.3)		45.6 (30.8–70.4)		48.2 (30.0–66.3)
Ischemic burden (mV-sec)								
Inferior (II) territory		–66 (–118 to –26)		–52 (–149 to –21)		–641 (–767 to –504)		–615 (–820 to –473)
Anterior (V2) territory		–66 (–142 to –16)		–50 (–124 to –13)		–597 (–859 to –435)		–632 (–905 to –489)
Lateral (V5) territory		–37 (–104 to –8)		–43 (–85 to –18)		–604 (–811 to –429)		–586 (–790 to –412)
Sum (II + V2 + V5)		–189 (–382 to –90)		–188 (–340 to –112)		–1,930 (–2,306 to –1,541)		–1,934 (–2,391 to –1,575)
Abbreviations: LFn, low frequency–normalized; pNN50, percentage of successive RR intervals that differ by > 50 msec; SDNN, standard deviation of RR intervals. Data are mean ± SD, or median (interquartile range). LFn and HFn are normalized units to account for variation in the total power and very low-frequency components. LF and SDNN reflect mainly sympathetic nervous stimulation; HF, pNN50, and RMSSD reflect parasympathetic tone. *p < 0.05 from Wilcoxon matched pairs signed rank test or Student’s paired *t*-tests as appropriate, mask versus no mask.								

During the 2-hr prescribed city center walks, subjects walked a comparable distance (6.37 ± 1.44 km vs. 6.40 ± 1.51 km), at a similar average speed (4.25 ± 0.96 km/hr vs. 4.27 ± 1.01 km/hr), and expended the same amount of energy [2.32 ± 0.52 metabolic equivalent tasks (METs) vs. 2.33 ± 0.55 METs] between visits when the face mask was or was not used. Despite this similar workload, mean ambulatory arterial blood pressure was significantly lower (93 ± 10 mmHg vs. 96 ± 10 mmHg) when the face mask was used, although heart rate was similar (Table 3). During the 2-hr walk, heart rate variability [high-frequency (HF) power, high-frequency normalized power (HFn), HF:low-frequency (LF) ratio, and root mean square successive differences (RMSSD)] was higher when wearing the face mask (Table 3). There were no significant differences in overall 24-hr ambulatory blood pressure or heart rate variability. There were no significant differences in the incidence of arrhythmias between the two visits (Table 4).

**Table 4:** Cardiac arrhythmias during 24-hr electrocardiographic monitoring periods among 98 coronary heart disease patients according to face mask use.

No. patients			Median no. of events per patient (interquartile range)		
Arrhythmia	Mask	No mask	Mask	No mask
Dropped beat		2		2		1 (1–1)		1 (1–1)
VT		1		2		2 (2–2)		1 (1–1)
Salvo		1		2		4 (4–4)		2 (1–3)
Bigeminy		15		18		4 (1–33)		6 (2–36)
Triplet		1		0		11 (11–11)		0 (0–0)
Couplet		9		4		2 (1–5)		2 (1–10)
Bradycardia		20		19		52 (3–275)		89 (9–347)
SVT		2		5		1 (1–1)		1 (1–1)
Trigeminy		3		4		12 (6–134)		10 (6–230)
Premature aberrant		79		77		17 (3–122)		22 (3–181)
Isolated aberrant		52		54		3 (1–16)		5 (1–25)
Premature normal		81		84		12 (3–56)		12 (3–42)
Abbreviations: SVT, supraventricular tachycardia; VT, ventricular tachycardia. Bradycardia defined as heart rate < 50 bpm. *p* > 0.05 for all using chi-squared (number of patients) and Mann–Whitney *U*-tests (number of events).								

## Discussion

PM air pollution is a major public health concern and is associated with increases in cardiovascular morbidity and mortality. In this study, we demonstrated that reducing personal exposure to urban airborne PM by means of a simple face mask is associated with a reduction in self-reported symptoms and improvements in objective measures of myocardial ischemia, blood pressure, and heart rate variability in patients with coronary heart disease. Reducing personal exposure to PM air pollution has the potential to reduce the incidence of cardiovascular events in patients with coronary heart disease living and working in industrialized or urban environments.

Using a robust PROBE design, we conducted a randomized controlled trial to assess the impact of reducing personal air pollution exposure in patients with coronary heart disease in a polluted urban environment. Through the use of portable monitoring devices and sample collection, we completed a detailed characterization of air pollutant exposure that demonstrated the remarkably complex and toxic composition and extremely high prooxidative potential of ambient air PM in Beijing. We combined individualized pollution monitoring with a comprehensive cardiovascular assessment that incorporated hemodynamic and electrophysiological monitoring in conjunction with GPS tracking. Despite reducing exposure only for a 48-hr period in patients chronically exposed to a polluted urban environment, we observed evidence of consistent beneficial effects on a range of biomarkers of cardiovascular health after the introduction of this simple but highly efficient face mask intervention.

*Myocardial ischemia.* In a cohort of 20 men with stable asymptomatic coronary disease, we previously demonstrated greater exercise-induced maximum ST segment depression during exposure to diesel exhaust (Mills et al. 2007). However, although acute air pollution exposure exacerbates myocardial ischemia, many persons around the world are chronically exposed to high levels of air pollution, and it is unknown whether interventions targeted at reducing exposure will decrease myocardial ischemia.

In the present study, we showed that decreasing personal exposure to ambient air pollution reduces maximal ST segment depression over a 24-hr period in patients with coronary heart disease. The significance of silent myocardial ischemia is still debated, but it has been associated with major cardiac events in the general population (Fleg et al. 1990). Moreover, in patients with recent myocardial infarction or unstable angina, the occurrence of silent ischemia is a poor prognostic factor and is associated with a significant increase (relative risk ~ 3–4) in major cardiac events and death (Cohn et al. 2003). It seems plausible, therefore, that the modest reduction in silent myocardial ischemia seen in this study might, if sustained, result in significant reductions in major cardiac events and cardiovascular mortality.

*Blood pressure.* Chronic exposure to air pollution is associated with increases in blood pressure in large epidemiological studies (Auchincloss et al. 2008). Similarly controlled exposure to concentrated ambient PM and ozone in healthy volunteers results in an acute increase in diastolic blood pressure (Urch et al. 2005). Hypertension is a major risk factor for atherosclerosis, and acute increases in blood pressure may trigger plaque rupture leading to an acute cardiovascular event. Consistent with this, exercise-related increases in blood pressure are predictive of the incidence of myocardial infarction (Mundal et al. 1996), stroke (Kurl et al. 2001), and cardiovascular mortality (Kikuya et al. 2000).

We recently reported that use of a face mask that decreased personal PM air pollution exposure reduced systolic blood pressure in healthy volunteers during a 2-hr walk by 7 mmHg (Langrish et al. 2009). The more modest 3-mmHg difference in mean arterial blood pressure after a 2-hr walk observed in the present study may be explained at least in part by the lower workload during walking in this older population with heart disease (estimated energy expenditures of 2.32 METs vs. 3.61 METs in the previous study population), coupled with the modifying effects of antihypertensive medications (Barclay et al. 2009), which were used by most of the present study population. However, interventional trials of blood pressure reduction suggest that even modest changes in blood pressure would reduce the incidence of major cardiovascular events at the population level (Williams 2005).

*Heart rate variability.* Heart rate variability is a reflection of the autonomic control (a balance of the sympathetic and parasympathetic nervous systems) of the heart and is a measure of the variation in the RR intervals on a continuous electrocardiogram. A reduction in heart rate variability has been demonstrated in patients with a variety of pathophysiological conditions, including hypertension, heart failure, and diabetes mellitus (Task Force 1996). Indeed, reduced heart rate variability has been linked to increased cardiovascular mortality (Nolan et al. 1998), and a large number of studies link exposure to air pollutants with a reduction in heart rate variability (Brook et al. 2010).

In the present study, we have shown that reducing personal exposure to PM air pollution in patients with coronary heart disease is associated with an improvement in heart rate variability during exercise, based on general measures of variability and variability in specific frequency bands. In this study, the changes demonstrated were predominantly in the HF-power band, which is associated with changes in parasympathetic tone, and an improvement may suggest an increased contribution of parasympathetic (vagal) tone to heart rate control. In our previous healthy volunteer study (Langrish et al. 2009), heart rate variability also increased after the face mask intervention, but changes were seen predominantly in the LF-power band, suggesting effects on sympathetic nervous system control. We suggest that this difference (HF-power vs. LF-power changes) may be related to the high use of beta-blocker therapy (74% of patients) in the present study population, which is likely to blunt any effects of exposure on sympathetic tone. The clinical relevance of acute changes in heart rate variability is not clear, although it has been demonstrated that the higher the variability, the lower the cardiovascular mortality (Kikuya et al. 2000). We suggest that a sustained improvement in heart rate variability has the potential to improve patients’ prognosis and reduce the impact of air pollution on cardiovascular morbidity and mortality.

*Symptoms.* Patients perceived fewer self-reported symptoms, a reduction in effort of work, and lower background pollution levels when they wore the face mask. Although we observed no change in the occurrence of self-reported anginal symptoms, this is perhaps not surprising given that we recruited a highly selected population with stable coronary disease, without significant clinical angina, and who were maintained on optimal medical therapy.

*Limitations.* We chose a PROBE study design because we wanted to determine the acceptability of wearing a face mask, as well as its potential beneficial effects on both symptoms and objective measures of cardiovascular health. We recognize that a double-blind approach incorporating a sham mask would reduce the potential for subjective bias and would therefore be considered more scientifically robust (Smith et al. 2007). In addition, we acknowledge that such an intervention may be more readily accepted in Chinese and Asian societies, where use of face masks is commonplace because of concerns over airborne diseases, pollution, and even fashion, and furthermore, that this may have affected patients’ reporting of symptom improvement. However, even a sham mask will filter air pollutants to some degree (Langrish et al. 2009), and true blinding is difficult to achieve given that large differences in mask efficiency would be readily apparent to trial participants, and differences in mask design would be obvious to investigators. It would also be anticipated that the greater effort of breathing through a mask during exercise would lead to an increase in blood pressure rather than the reverse.

We have assessed an acute intervention, and it remains to be seen whether wearing a face mask for more prolonged periods would have sustained benefits that could affect clinical outcomes.

## Conclusions

In this randomized controlled crossover intervention trial, we observed that reducing personal exposure to PM air pollution was associated with small but consistent improvements in objective measures of myocardial ischemia, exercise-related increases in blood pressure, and heart rate variability in patients with coronary heart disease. Although efforts to reduce emissions are critical to reducing exposures to the population as a whole, use of a face mask may be an effective individual-level intervention for high-risk populations. The use of a face mask has the potential to reduce the incidence of acute cardiovascular events, as well as improving patients’ general well-being, particularly in developing countries where pollutant exposures are high and resources to reduce emissions are limited.

## Supplemental Material

(3.4 MB) PDF
