# Supplementary material for: Reducing Personal Exposure to Particulate Air Pollution Improves Cardiovascular Health in Patients with Coronary Heart Disease
Source: Environ Health Perspect. 2012 Jan 3;120(3):367–72. doi: 10.1289/ehp.1103898 (PMC3295351; doi:10.1289/ehp.1103898)

Reducing Personal Exposure To Particulate Air Pollution Improves Cardiovascular Health In Patients With Coronary Heart Disease

*Jeremy P Langrish[1] and *Xi Li,[2] Shengfeng Wang,[2] Matthew MY Lee,[1]
Gareth D Barnes,[1] Mark R Miller,[1] Flemming R Cassee,[3]
Nicholas A Boon,[1] Ken Donaldson,[1] Jing Li,[4] Liming Li,[2]
Nicholas L Mills,[1] David E Newby,[1] Lixin Jiang[4]
*contributed equally to this manuscript.

[1]Centre for Cardiovascular Science, University of Edinburgh, United Kingdom
[2]Department of Epidemiology & Biostatistics, School of Public Health, Peking University, Beijing, People's Republic of China
[3]National Institute for Public Health & the Environment, Centre for Environmental Health Research, Bilthoven, The Netherlands
[4]Fuwai Hospital & Cardiovascular Institute, Chinese Academy of Medical Sciences & Peking Medical Union College, Beijing, People's Republic of China

Correspondence and requests for reprints:
Dr Jeremy Langrish
University of Edinburgh
Centre for Cardiovascular Science
Room SU.305, Chancellor's Building
49 Little France Crescent
Edinburgh
EH16 4SB
United Kingdom
Tel: +44 131 242 6428
Fax: +44 131 242 6379
Email: jeremy.langrish@ed.ac.uk

Short title: Reducing Personal Air Pollution Exposure and Cardiovascular Health

Trial registration: http://www.ClinicalTrials.gov NCT00809653

Table S1. Particle characteristics and chemical composition for particulate matter collected in Beijing China in March 2009. Data is expressed as mean concentrations (< represents result below lower limit of detection). Health and the Environment, Bilthoven, Netherlands].

**Particle Characteristics in Beijing**		**Beijing** *17–18th March 2009*		
		**Coarse**	**Fine**	**Ultrafine**
		$PM_{2·5 – 10}$	*$PM_{0·18–2·5}$*	*$PM_{<0·18}$*
Mass, µg/m^3 (% total mass)		114 (34%)	151 (45%)	72 (21%)
**Carbon**	Total carbon (µg/mg dust)	166	497	571
	Elemental carbon (µg/mg dust)	12	91	99
	Organic carbon (µg/mg dust)	154	445	472
	EC : OC ratio	0·08	0·21	0·21
**Anions**	Nitrates (µg/mg dust)	93	590	510
	Sulphates (µg/mg dust)	77	217	183
**16 US-EPA Priority Polycyclic Aromatic Hydrocarbons (PAHs)**	Napthalene (ng/g dust)	2599	3015	4265
	Acenaphthylene (ng/g dust)	2107	1771	7315
	Acenaphthene (ng/g dust)	3540	2882	3886

	Flourene (ng/g dust)	3234	2558	3399
	Phenanthrene (ng/g dust)	1959	3004	3502
	Anthracene (ng/g dust)	1423	1100	1553
	Fluoranthene (ng/g dust)	1649	17858	16071
	Pyrene (ng/g dust)	1780	16689	14485
	Benzo[a]anthracene (ng/g dust)	1728	9501	7343
	Chrysene (ng/g dust)	2323	22107	21533
	Benzo[b]fluoranthene (ng/g dust)	2235	27478	29031
	Benzo[k]fluoranthene (ng/g dust)	1321	22251	18664
	Benzo[a]pyrene (ng/g dust)	1781	18099	17762
	Ideno[123–cd]pyrene (ng/g dust)	1573	16378	15061
	Dibenzo[ah]anthracene (ng/g dust)	2108	3510	3359
	Benzo[ghi]perylene (ng/g dust)	1624	24488	24829
**Hopanes**	17α(H),21β(H)22,29,30–Trisnorhopane (ng/mg dust)	1·9	2·8	17·1

	17α(H),21β(H)–Hopane (ng/mg dust)	4·4	3·7	10·3
**Steranes**	20R–5α(H),14β(H),17β(H)–Cholestane (ng/mg dust)	3·2	1·8	24·7
	20R–5α(H),14α(H),17α(H)–Cholestane (ng/mg dust)	0·0	1·3	14·7
	20R–5α(H),14β(H),17β(H)–24S–methycholestane (ng/mg dust)	0·0	1·2	12·2
	20R–5α(H),14β(H),17β(H)–24R–Ethylcholestane (ng/mg dust)	1·9	1·5	15·4
**Heavy Metals**	Aluminium (ng/mg dust)	18733	4093	7486
	Antimony (ng/mg dust)	64	71	75
	Barium (ng/mg dust)	2403	678	968
	Cadmium (ng/mg dust)	8	30	27
	Calcium (ng/mg dust)	64357	12854	20119
	Cerium (ng/mg dust)	50	12	17
	Chromium (ng/mg dust)	78	60	58
	Copper (ng/mg dust)	303	292	313
	Iron (ng/mg dust)	38976	11045	14001

	Lanthanum (ng/mg dust)	25	5	9
	Lead (ng/mg dust)	228	1364	1277
	Magnesium (ng/mg dust)	18799	3791	6293
	Manganese (ng/mg dust)	847	629	665
	Neodymium (ng/mg dust)	20	4	6
	Nickel (ng/mg dust)	85	57	70
	Potassium (ng/mg dust)	8211	17556	17246
	Silicon (ng/mg dust)	2396	1659	2669
	Sodium (ng/mg dust)	9475	5060	5679
	Strontium (ng/mg dust)	326	85	125
	Sulphur (ng/mg dust)	13060	33606	32504
	Titanium (ng/mg dust)	970	326	402
	Vanadium (ng/mg dust)	54	32	31
	Zinc (ng/mg dust)	888	2542	2689

Figure S1. Prescribed walks from the Fuwai Hospital (west of city centre in Xicheng District) and ChaoYang Hospital (east of city centre in ChaoYang District). Map freely available online from Weller Cartographic Services Ltd. at http://www.mapmatrix.com/tmhtm/mapcat.html#Asia%20Catalogue

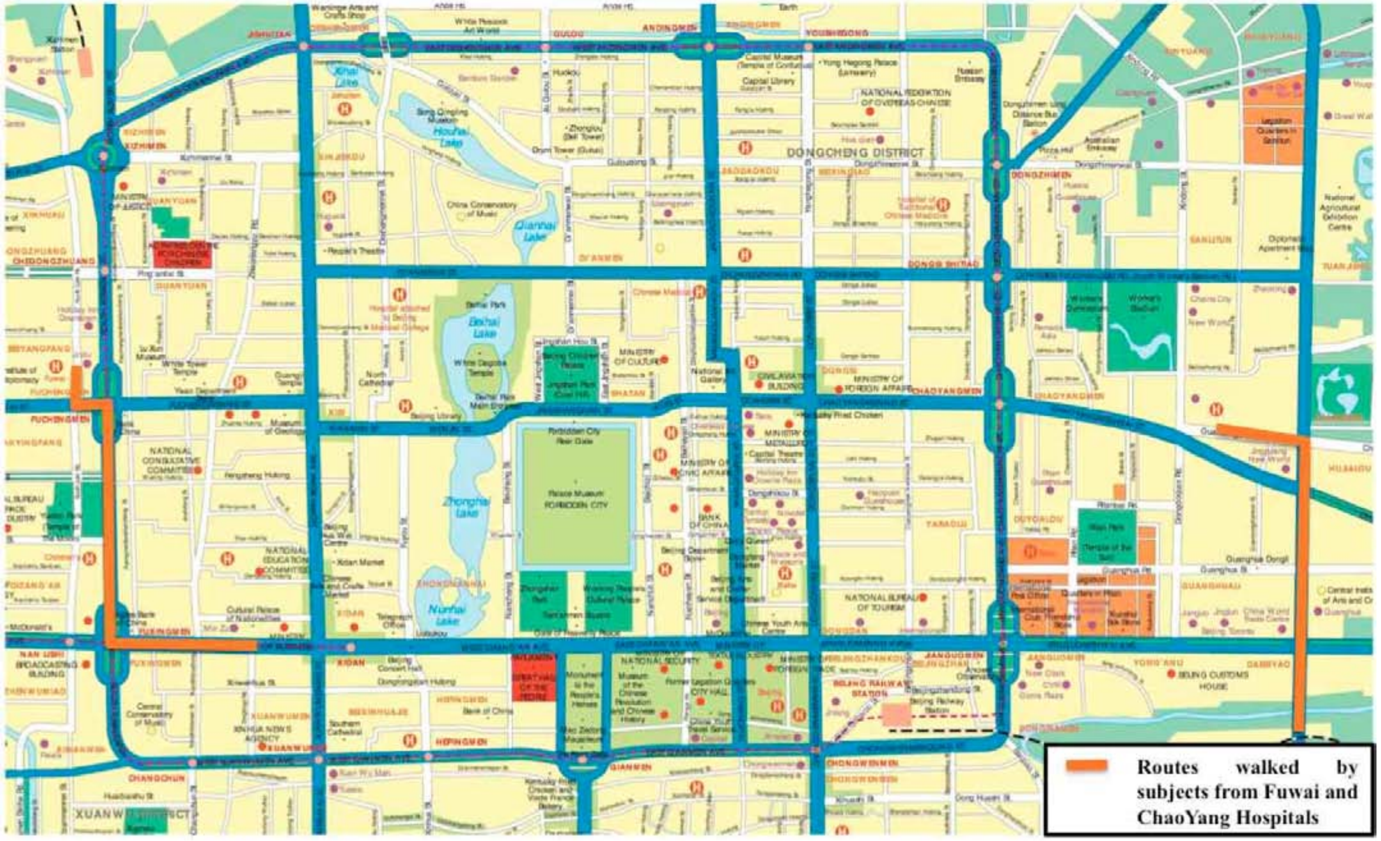

Supplement: (3.4 MB) PDF [file ehp.1103898.s001.pdf]
